# An Integrated Approach to an Emerging Problem: Implementing a Whole Year of Camera Trap Survey in Evaluating the Impact of Wildlife on Tick Abundance

**DOI:** 10.1155/2024/4064855

**Published:** 2024-09-26

**Authors:** Ezio Ferroglio, Rachele Vada, Flavia Occhibove, Mattia Fracchia, Federica De Cicco, Pablo Palencia, Amir Reza Varzandi, Stefania Zanet

**Affiliations:** Department of Veterinary Sciences University of Turin, Largo Paolo Braccini 2, Grugliasco 10095, Italy

## Abstract

Tick-borne zoonoses are an emerging health issue. The expansion of ticks is mainly driven by climatic changes but also by new approaches to the management of the natural environment, increasing the abundance of vertebrate host species and thus the potential exposure to tick bites for both humans and companion animals. In this context, a holistic approach to studying ticks' ecology is required. In the present work, we shed light on the link between environmental tick abundance (global and specific of *Ixodes ricinus* nymphs, as the highest zoonotic threat) and the temporal occupancy of wildlife host species retrieved from camera traps (namely, wild ruminants, mesocarnivores and wild boar). We modelled this relationship by integrating abiotic factors relevant to tick survival, such as the vegetation cover and saturation deficit, and estimated the accuracy of prediction. To collect these data, we deployed camera traps in a peri-urban Natural Park in Northwest Italy to monitor wildlife for 1 whole year while collecting ticks in front of camera traps by dragging transects every 2 weeks. Overall, wildlife temporal occupancy showed an additive impact on tick abundance for species that are preferential hosts (deer and mesocarnivores) and a detractive impact for wild boar, which also presented a lower tick burden, particularly with regard to the tick species collected in the environment (mainly *I. ricinus* and *Haemaphysalis punctata*). Accuracy of prediction was higher for *I. ricinus* nymphs rather than the global model. Temporal fluctuations in the tick population were also highlighted. Wildlife temporal occupancy was not constant and varied between seasons according to feeding habits. In conclusion, we highlighted the utility of camera trap data to investigate tick ecology and acarological risk. This information is crucial in informing monitoring and prevention strategies to decrease the risk of tick bites in humans and thus zoonotic risk of tick-borne diseases.

## 1. Introduction

Tick-borne zoonoses (TBZs) including Lyme disease, babesiosis and tick-borne encephalitis are currently considered an emerging problem in several European countries [1–4]. Ticks are indeed expanding their altitudinal and latitudinal range, mainly due to temperature increase and loss of seasonality [5–8], being climatic and environmental factors the main drivers of tick survival and activity pattern [9, 10].

In addition, anthropogenic factors influencing wildlife populations are also impacting the trends of ticks' expansion [6]: reforestation and land abandonment have increased wildlife abundance after a population contraction, particularly for deer species, yielding to ecological conditions conducive for the re-emergence of ticks [11, 12]. Animal welfare politics moving towards extensive animal breeding, increase of wildlife management, habitat destruction and popularity of outdoor sporting are among the several reasons that are bringing wildlife closer to urbanized areas, people and their animals [13, 14]. In sympatric environments for wildlife, domestic animals and people, a holistic approach is fundamental to understanding the mechanisms impacting the presence and abundance of questing ticks; this can be accomplished only by integrating high-quality wildlife population data with biotic and abiotic environmental information.

The debate about how to reduce tick abundance is still open [15]. On a small scale (house yards for instance), chemical and biological methods have been experimented, without a definitive success [16, 17]. A review of their pitfalls and potentialities is presented by Ostfeld et al. [18]. Those methods are impractical at a large scale; therefore, in the wider natural environment, other options have been explored. For instance, vertebrate host reduction was tested on deer populations, but ultimately, it did not provide the expected results and still needs further confirmation [19–21]. On a small scale, integrated pest management actions have been proven to be highly effective [22], and this seems to be a suitable direction for the natural environment as well. There is much interest in integrating data from wild hosts, the environment and human activities to identify risk areas for tick bites on people (e.g. [23]).

With an increasing trend in the last few years, camera traps have been claimed by the scientific community to be the most efficient tool to monitor wildlife in terms of cost-effectiveness and reliability of results [24]. They also present the advantage of providing high-resolution data with limited wildlife disturbance, allowing us to gather information that would be impossible to obtain otherwise. Worldwide, few examples have been produced of use of camera trap data to study the relationship between population composition and tick abundance [25–30]. In some cases, they have been examples of an integrated approach including small mammals [31, 32]. Hofmeester et al. [28] implemented camera traps to relate wildlife passage rate to tick abundance, highlighting a positive correlation and introducing camera trap data to the study of the relationship between densities of questing ticks and the availability of different vertebrate species. In this work, we aim to analyse the impact of wildlife temporal occupancy (TO) (i.e. the amount of time a species is seen in a given site) on questing ticks abundance, considering also abiotic factors and fluctuations in time. The information gathered might inform policy and practice to prevent and control transmission of relevant TBZs.

## 2. Materials and Methods

### 2.1. Study Area and Objectives

This study was implemented in La Mandria Natural Park, Northwest Italy, where we performed a 1-year-long data collection, aiming to include the seasonal variation in the analysis. La Mandria Natural Park is a fenced park of 6571 ha at a mean altitude of 386 ma.s.l. (±269 m), mostly covered in deciduous forest and fields of grassland for mowing. The annual precipitation in 2020 was 898.2 mm, and it presented an average temperature of 13.25°C. It welcomes an average of 2000 visitors every day, and wildlife management is constant throughout the year. Few horse farms and cultivated areas are present inside the park. Wild ungulate density for those areas was studied in the framework of the European Wildlife Observatory [33]. The highest densities were obtained for wild boar (*Sus scrofa*), 15.26 ± 2.41 individuals/km^2^ (with confidence intervals), and red deer (*Cervus elaphus*), 11.03 ± 3.17 individuals/km^2^, followed by fallow deer (*Dama dama*), 3.31 ± 1.16 individuals/km^2^, and roe deer (*Capreolus capreolus*), 1.90 ± 0.97 individuals/km^2^. Those densities were obtained by implementing the random encounter method [34], which allows to estimate the density of those species without the need of individual recognition, and contextually sampling for the present work.

Detailed objectives for the present study were to (i) determine the relationship between tick abundance and wildlife TO, considering abiotic factors and, especially, temporal fluctuations; (ii) detail the above relationship for *I. ricinus* nymphs only, as the developmental stage and species posing the highest zoonotic threat; (iii) identify variation in temporal occupancy for wild species monitored through camera traps; and (iv) quantify tick burden for culled wildlife in the park.

### 2.2. Sampling Design

**Figure 1:**
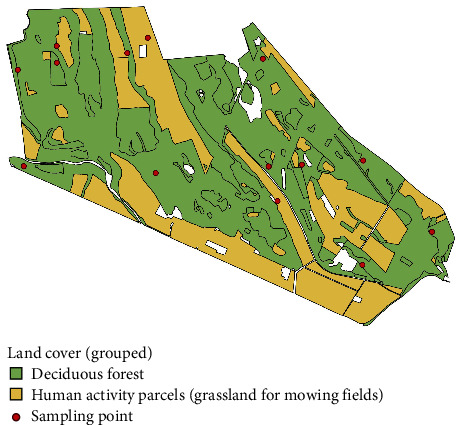
Map of the study area with the CLC categories: yellow for human activity parcels (grassland for mowing) and green for deciduous forest. Sampling points are indicated as red dots.

Information on land cover was retrieved from CORINE Land Cover (CLC), available for the study area from Geoportale Piemonte (https://www.geoportale.piemonte.it/cms/progetti/land-cover-piemonte). CLC (Corine Land Cover Piemonte level) was reclassified into two macro types of vegetation: deciduous forests and human activity parcels (fields of grassland for mowing). We identified an adequate number of sampling points to cover the area homogenously, randomly placed with respect to animal movement and proportionally representative of different macro types of vegetation. This led to a total of 14 sampling points in the study area (Figure 1). In each sampling point, a camera trap (either Browning Force Edge—Model BTC-7E, or Browning Dark Ops Apex—Model BTC-6HD-APX) was deployed facing north, 50 cm above the ground, with the sensor angled parallel to the slope. Cameras were set to be operative all day, to record a burst of eight consecutive pictures (rapid fire setting) at each activation, with the minimum time laps (0.22 s) between consecutive activations. Nocturnal pictures were illuminated with infrared flash (low glow). Neither baits nor attractants were used. The date and time of each capture were automatically stamped onto each picture.

**Figure 2:**
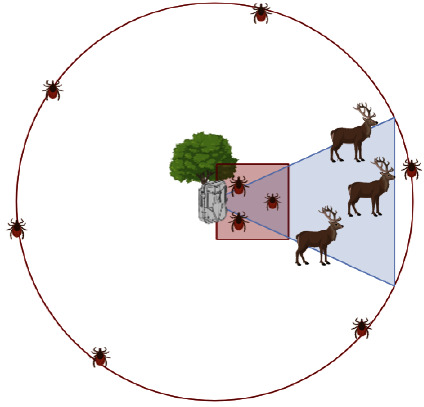
Graphical representation of the sampling design, including tick dragging transects, referred to the camera trap deployment and field of view. Animals that are in the field of view are recorded by the camera trap, while a 100 m^2^ (10 × 10 m) square dragging transect is performed in front of it, and a circle of 26 m radium dragging transect is performed around the camera trap.

Contextually with the camera trap placement, two tick dragging transects were performed on site: a 10 m^2^ square in front of the camera trap (being 10 m the furthest distance at which data could be obtained with almost perfect detectability of animals) and in a circle of 26 m radium and centre in the camera trap (the maximum field of view of the camera trap), to sample the whole area around the site. Ticks collected from dragging were stored in ethanol 70% and later identified by morphological keys [35–37]. A graphic representation of the transects is presented in Figure 2. Together with camera traps, we placed a sensor (temperature and humidity data logger RS PRO, USB) to register hourly the temperature and humidity 15 cm above the ground.

Camera traps were checked to retrieve SD cards and control batteries every 2 weeks for 1 year (August 2020 to August 2021), and, on the same occasion, the tick-dragging transects were repeated. We performed a total of 26 repetitions during the whole study.

### 2.3. Dataset Preparation

For statistical purposes, we considered an observation as the data collected at each sampling point and repetition. Therefore, each observation was characterized with (a) tick abundance, (b) wildlife temporal occupancy (TO), (c) mean normalized difference vegetation index (mean NDVI), (d) maximum saturation deficit (maxSD, following Perret et al. [38]) and (e) month. Data was prepared as follows:

i. Tick abundance. Tick counts from both transects were then summed by sampling point and repetition, and the abundance was calculated referring to the area covered by both transects.
ii. Wildlife TO. We extracted TO for wild species with good detectability on camera traps, namely, wild boar, red deer, roe deer, fallow deer, red fox (*Vulpes vulpes*), badger (*Meles meles*), pine marten (*Martes martes*) and beech marten (*Martes foina*). Camera trap pictures were analysed to extract, for each individual: sampling point, date of passage and time spent in front of the camera (calculated as the number of seconds between the first picture and the last). As the aim of this analysis was to calculate the species TO, no individual recognition was required, and, for simplicity, an individual exiting the field of view and entering it again was considered as a new individual, so that only the actual time spent in front of the camera trap was considered. In case that more than one individual appeared in the picture, the time of each animal was calculated separately and eventually summed. We summed the seconds for each species per sampling point and repetition. For statistical purposes, we merged seconds for all deer species and mesocarnivores (red fox and mustelids), so that three TO data types were implemented in the analysis: wild boar, wild ruminants and mesocarnivores.
iii. Mean NDVI. These data were retrieved with the R [39] *MODIStsp* package [40] at a resolution of 250 m and 16-day intervals. Dates were matched with sampling dates and point coordinates, and the mean was calculated per sampling point and repetition.
iv. MaxSD. With the sensor we registered, for each sampling point, the hourly mean temperature and humidity and calculated the daily mean SD [41] per sampling point, from which we derived the maxSD per sampling point and repetition. Mean and minimum SD were considered but ultimately discarded, based on the high collinearity with max SD and being the max SD the most limiting factor to tick activity [38].

### 2.4. Statistical Analyses

We described the time fluctuations in ticks' abundance and tested abundance correlation among stages (Spearman's rank correlation). Also, we tested different abundance between the two CLC categories with a Kruskal–Wallis test.

We modelled the impact of six explanatory variables (max SD, mean NDVI, TO for wild boar, wild ruminants and mesocarnivores and month) on tick abundance (global model) and, as a second step, on *I. ricinus* nymph abundance. We proceeded alike for both models. We excluded outliers and months that, for tick ecology reasons, returned no ticks, thus creating a bias towards zero values not determined by wildlife presence (November, December and January). We also checked data for normality and applied to each variable a normalization transformation if needed, with the *bestNormalize* package [42] in R [39]. Relationships among explanatory and response variables were modelled using general additive mixed models (GAMMs) [43]. GAMMs extend generalized linear models (GLMs/GLMMs) by using smooth functions to define nonlinear relationships between the response and explanatory variables and by combining predictor variables additively [43]. This approach is particularly useful to better describe relationships in ecological systems. Non-linear relationships are expressed as “smooths”, which are evaluated by visual inspection of a plot rather than evaluation of coefficients, as done in linear regression [43]. We tested the performance of the variables as linear predictors and smooth terms and, however, expected a linear relationship between maxSD [38] or mean NDVI and tick abundance and a non-linear relationship for month [9] and wildlife TO. We also tested the effects of the interaction between different variables. We tested spatial random effects for the sampling point. Moreover, we explored different variance weights, including by sampling point, by month and by season and paired interactions between them. We explored different variable interactions for linear and non-linear predictors, as well as variance weight combinations. Model selection was based on three parameters: (i) the statistically significant (ANOVA test) lowest Akaike information criterion (AIC), (ii) a satisfactory *R*^2^ (considering the complexity of the biological phenomena observed) and (iii) good performance of residuals analysis and smooth graphs. Analysis was performed in R [39] with the function *gam* of the *mgcv* package [43]. In addition to *R*^2^, to evaluate the predictivity of the models, we split our dataset in train (70% of the observations homogeneously through seasons) and test (30% of the observations). We defined our model on the train dataset and predicted values on the test dataset. We compared the test observed and predicted values with Spearman correlation and the mean absolute error (MAE).

After analysing the impact of wildlife TO on tick abundance, we also described the differences in wildlife TO in relation to CLC classes and seasons using, due to non-normality, the Kruskal–Wallis non-parametric test of difference.

### 2.5. Tick Collection on Culled Wildlife

To evaluate the tick burden on wildlife and get a more complete picture of the study area, we sampled the carcases culled by park rangers for 6 months (December to June). Only wild boar and red deer were available for the purpose, as both species are culled for numerical control throughout the whole year. We collected ticks and stored them in ethanol for morphological identification as for questing ticks. A descriptive analysis was performed to identify tick load and tick species composition on sampled wildlife, and a Mann–Whitney test was performed to identify the difference in tick burden between the two animal species.

## 3. Results

### 3.1. Tick Transects and Camera Trap Pictures

We registered a total of 300 observations (the data collected at each sampling point and repetition, as the combination of ticks, TO calculation and environmental variable detection). Almost 80% of collected ticks belonged to larval stages. The most abundant genera were *Ixodes* (*I. ricinus*), which made 63% of collected ticks, and *Haemaphysalis* (*H. punctata* and *H. concinna*), which made 36% of collected ticks (Table 1).

**Figure 3:**
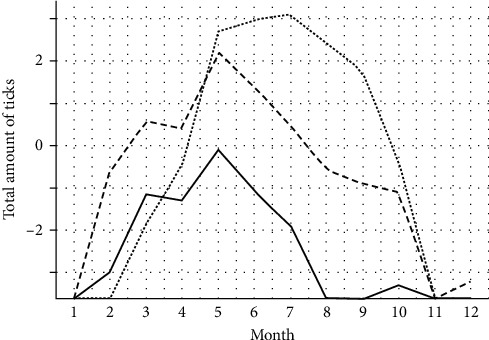
Temporal trend of ticks collected in La Mandria. The graph shows the trends per developmental stage, plotting the logarithmic mean number over the month of collection: solid line, adults; dashed line, nymphs; and dotted line, larvae.

Ticks presented a different seasonal trend depending on the life stage, with a peak between the end of spring and the beginning of summer and a second peak for larvae in August (Figures 3 and S1 for species detail). Larval abundance exhibited a significant positive correlation with nymphal abundance (*p* < 0.05) but not with adult abundance (*p* > 0.05). Additionally, nymphal and adult abundances were also significantly correlated (*p* < 0.05).

Tick abundance in grassland for mowing fields was lower (*p* < 0.05) than in deciduous forest.

Regarding wildlife TO, we registered the highest for wild boar (706,688 s in the whole study period) and red deer (108,938 s), followed by fallow deer (58,017 s) and roe deer (5437 s). All ungulates were recorded in all sampling points, with the exception of roe deer, which was only recorded in 11 out of 14 sampling points. Mesocarnivores were recorded in all sampling points with a total of 7524 s.

### 3.2. Temporal Occupancy Model

Following the best model selection through *R*^2^, AIC and residual analysis (Table S1 and Figure S2), tick abundance in both models was best predicted by maxSD and meanNDVI as linear predictors, month as a non-linear predictor and the interaction of TO for mesocarnivores, wild boar and wild ruminants with season as non-linear predictors. The models, based on a Tweedie distribution [44], implemented the sampling point as a random variable and weighted variance by the interaction of the sampling point and month. All linear predictors and smooth terms were statistically significant (Table S2 for coefficients).

**Figure 4:**
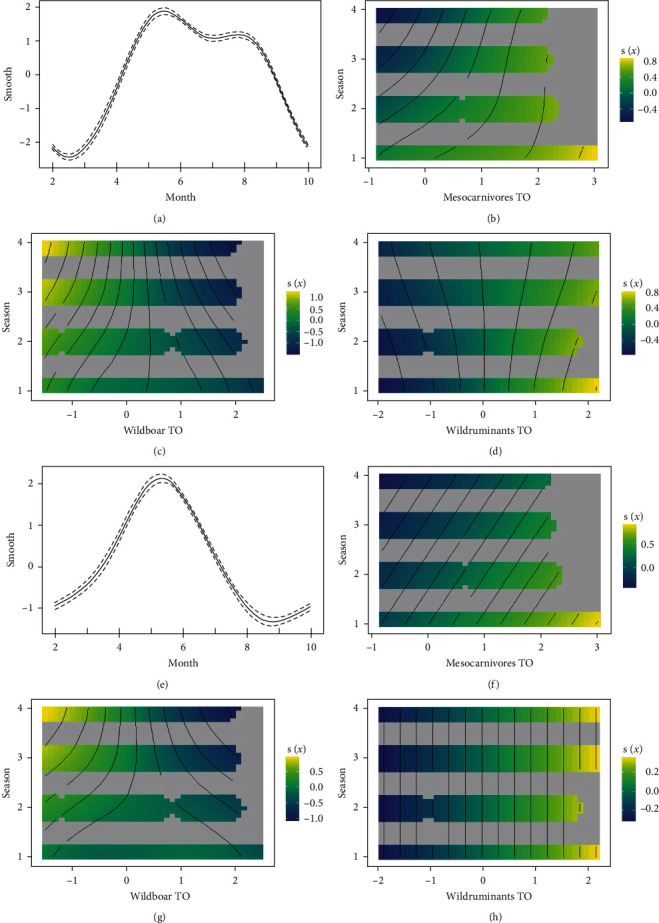
2D smooth effect plots. (a–d) Global model. (e–h) *I. ricinus* nymph model. (a and e) Month effect on tick abundance. Smooth effects on tick abundance are presented by season, for mesocarnivore (b and f), wild boar (c and g) and wild ruminant (d and h) TO. Season codes are 1 (summer), 2 (autumn), 3 (winter) and 4 (spring). TO ranges from negative to positive values due to data transformations. TO for mesocarnivores and wild ruminants shows an additive effect (light green–yellow) for most seasons and a detractive effect (dark green–blue) at low TO. The increase in wild boar TO shifts its effect to being detractive to tick abundance.

The variable effect was similar for global and *I. ricinus* nymph model. The smooth function for the variable 'month' reproduced, in both models, the peaks of the raw trend (Figure 4a and 4e). TO for mesocarnivores shows an additive effect for most seasons and a detractive effect at low TO during spring (Figure 4b and 4f). Similar results were obtained for wild ruminants, but the trend was constant through all seasons, with a detractive effect at low TO and an additive effect at high TO (Figure 4d and 4h). As for wild boar, TO increase shifts its effect to being detractive to tick abundance, with a marked detractive effect at higher TO and an additive effect at lower TO during spring (Figure 4c and 4d).

In the global model, the deviance explained was of 39%, Spearman correlation between predicted and observed data of 0.36 (*p*=0.003) and MAE of 15. *I. ricinus* nymph model returned 39.1% deviance explained; Spearman correlation between the predicted and observed data was of 0.44 (*p*=0.0003) and a MAE of 1.93.

### 3.3. Variations of TO

The only wild ruminant displaying different TO was red deer, which seemed to spend more time in deciduous forests during summer and winter. Wild boar spent more time in deciduous forests during summer and in parcels with human activity (fields of grassland for mowing) during spring, which corresponded to the habitat where mesocarnivores spent more time in winter.

### 3.4. Tick Collection on Culled Wildlife

**Figure 5:**
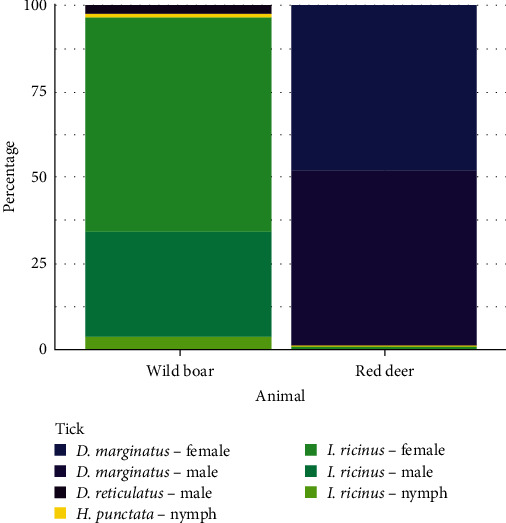
Percentage of tick individuals per species and life stage over the total number of tick collected.

We sampled 145 animal carcases culled by park rangers, 24 red deer and 121 wild boar, for half a year (December to June). Of these, 22 red deer (91.67% ± 11.06%, 95% confidential interval) and 42 wild boar (34.71% ± 8.48%) were infested by ticks. *D. marginatus* was the main species found on wild boar (38.84% ± 8.14% of individuals had at least one *D. marginatus* tick), while *I. ricinus* was for red deer (75% ± 17.03% of individuals with at least one *Ixodes* tick), as shown in Figure 5.

At least one individual for each tick genus was found on both ungulates (Table 2). Most ticks were adult (either male or female), but a nymph of *H. punctata* was found on both ungulate species, and three nymphs of *I. ricinus* were found on red deer. Through a non-parametric statistical test (Mann–Whitney test), we identified a significant difference in parasitic load between the two species for *Ixodes* spp.

## 4. Discussion

Camera trap data allowed us to model the impact of wildlife TO on questing tick abundance, factoring in also other biotic and abiotic factors. We highlighted that, for the preferential hosts of the collected tick species (mesocarnivores and wild ruminants [45]), the increase in TO changed its effect with a positive trend, with animals removing ticks at low TO and having an additive effect when increasing TO. On the contrary, wild boar, which was less frequently parasitized by *I. ricinus* and *Haemaphysalis* spp. [45], showed an opposite trend, with a detractive or a null effect in most cases. These results were in accordance with the ones obtained in previous studies with camera trap data, showing the positive impact of deer species on tick abundance [27, 28, 31]. In this case, we confirmed the necessity to merge all deer species (as in [31]), but we kept wild boar separate, obtaining quite opposite effect on questing tick abundance, which was, as already stated, justifiable by the general low parasitic burden of major species for this study. In relation to mesocarnivores (fox in particular), previous studies have detected a detractive impact on *I. ricinus*, while our results showed an opposite trend. Given that *I. ricinus* is frequently found on foxes too, epidemiological and habitat characteristics may have influenced the contradictory result.

The population structure of ticks and its temporal fluctuations, with peaks corresponding to months with temperature and humidity in the range of tick suitability, and scarce or even null presence in colder months, were consistent with previous studies [9, 38, 46, 47]. Sampling points in fields of grassland for mowing did return a low number of ticks, most likely because of the absence of shelter with low grass, which exposes ticks to more extreme temperatures and lower humidity, as well as scarce sensitivity of dragging transects (compared with other methods) with high grass, where the most abundant stage (larvae) quests closer to the ground [48].

In terms of tick species composition, *I. ricinus* was, as expected, the most abundant tick collected in the environment. On the other side, through dragging, we did not register *D. marginatus* in an area with high density of wild boar (which is among its preferential host species in continental and temperate climates [45]). Carcass sampling did return instead of several wild boar carrying *D. marginatus* (Figure 5). These findings might corroborate the speculation over the nidicolous status of larval and nymphal stadia for this genus [41], a behaviour that would lower the sensitivity of dragging transects and explain the low environmental presence despite the high parasitic load on wild boar. Moreover, culling activity may constrain a good sampling representability: in the park, 10 times more wild boar are culled than red deer, which are eventually little represented, and no roe deer, an important host for *I. ricinus* [49], is culled at all. *I. ricinus* is indeed a species that is found on wild boar with low frequency, as shown by the sampling of culled animals in the park and by literature [50–52].

Different tick stages have feeding preferences for different hosts [9, 53], and a broad range of small-animal species (from small mammals to birds) has been showed as the main host of immature stages for both *I. ricinus* and *H. punctata* [9, 54]. However, all stages have been found feeding on deer, although at different body locations [55, 56]. Therefore, we face a complex interaction between environmental factors and animal species that determines the abundance of ticks at different stages, as it has already been explored elsewhere (e.g. [57]). This complexity must be considered to explain our choice of preserving a global model: we addressed the impact of wild ungulates and mesocarnivores TO on the global population of questing ticks. For a finer scale work, information on ticks' life cycle such as moulting and egg hatching time in different environmental conditions would provide a useful reference. Additionally, the inclusion of small mammals would be another significant step forward to encompass hosts for immature tick stages and reservoirs for significant TBZs, such as Lyme disease [58].

We tested the model for *I. ricinus* nymph, as the species vector for most zoonotic tick-borne diseases [45] and the developmental stage that most frequently parasitizes humans [59]. Nymphs are considered the most important stage in transmitting Lyme disease and TBE to humans [60]. This is because in nature nymphs are much more numerous than adult ticks and because nymphs, compared to adult female ticks, are more easily overlooked due to their smaller size and less conspicuous colouration. Despite similar outputs for both models, the predictivity of the finer one was higher. Thus, due to different behaviours and ecology of tick species and developmental stages, breaking down the response variable allows us to obtain outputs with higher accuracy. The abundance of following stages (larvae with nymphs and nymphs with adults) was closely correlated, potentially explaining the similar outputs generated by the two models. In contrast, additional factors such as survival rates may have influenced the absence of correlation between adults and larvae.

Considering all habitat-related factors that may determine the presence of ticks in the environment, model predictivity was quite satisfactory and represented an indicator of the usefulness of camera trap data to define acarological risk. A step forward to a practical application of these results is the analysis of wildlife TO variability in the study areas according to different habitat types. Our findings were in accordance with previous studies about habitat selection, especially regarding wild boar preferences in different seasons (i.e. [61, 62]), probably following feeding and sheltering preferences. Understanding which sites are preferred by wildlife represents useful information to assess the risk of tick bites for people accessing the natural environment. However, it is important to consider that the modest extension of the study area and habitat and management conditions of natural areas impose limits to the generalizability of the study's findings. The identification of risk areas for ticks' presence and consequently TBZs transmission, entering itself into the broader approach of integrated management, is a promising but yet not much-explored field. However, a more detailed ecological analysis, including landscape analysis such as habitat fragmentation and connectivity, is needed to predict TO differences in different sites and therefore tick bite risk. As camera traps are getting more popular, for their efficiency in retrieving highly valuable data, deployment sites are increasing. In these areas, information retrieved from camera traps might be used to investigate the relationship between wildlife TO and tick abundance, identifying risk zones for tick bites and providing evidence-based information for effective decision-making in the context of public health.

## 5. Conclusions

In this study, we emphasise the impact and predictivity of time spent by wildlife in a specific site, extracted from camera trap data, on the abundance of ticks in the environment. This effect was mainly related to ticks' host preferences and seasonality. The information retrieved from camera traps was crucial in investigating ticks' ecology, demonstrating another application for this already useful tool. To properly achieve a holistic approach to assess the risk of tick bites in humans in natural areas, which is proving to be the best approaches to limit the spread of TBZs, more detailed environmental data are required, as well as small mammal populations data, traditionally considered important tick hosts and TBDs main reservoirs.

## Figures and Tables

**Table 1:** Total number of ticks collected with dragging, per developmental stage and species.

Developmental stages	Total amount of collected ticks
Larvae	2069
Nymphs	480
Adults	48

**Species**	

*Ixodes ricinus*	1631
*Ixodes hexagonus*	5
*Haemaphysalis punctata*	648
*Haemaphysalis concinna*	247
*Dermacentor reticulatus*	6
*Rhipicephalus sanguineus* complex	60

**Table 2:** Tick species distribution as average abundance of ticks per tick species found on red deer and wild boar, with 95% confidential interval.

	Red deer	Wild boar
*D. reticulatus*, adult male	0.17 (±0.33)	0
*D. marginatus*, adult male	0	0.83 (±0.41)
*D. marginatus*, adult female	0	0.78 (±0.46)
*I. ricinus*, adult male	2.08 (±1.24)	0
*I. ricinus*, adult female	4.25 (±2.88)	0.01 (±0.02)
*I. ricinus*, nymph	0.25 (±0.35)	0
*H. punctata*, nymph	0.08 (±0.16)	0.01 (±0.02)
No tick found	0.17 (±0.22)	0.72 (±0.08)

## Data Availability

The data that support the findings of this study are available from the corresponding author upon reasonable request.

## Supporting Information

Supporting Information Figure S1: Temporal trend of ticks collected in La Mandria. The graph shows the trends per developmental stage and species, plotting the logarithmic mean number over the month of collection. The red line defines all species together, while single species are plotted with thinner lines. Table S1: AIC model selection. Significance codes: *p*=0 ‘ ^*∗∗∗*^', *p*=0.001 ‘ ^*∗∗*^',*p*=0.01 ‘ ^*∗*^' and *p*=0.05‘.'. The *k* value is the smoothing parameters and determines how many basis functions are used to approximate the smooth function for each predictor. The cubic regression splines (‘cc') were used as basis function (bs). Figure S2: Residual analysis of the final GAM global model (A) and *I. ricinus* nymph model (B). We plot the following: (top-left) deviance residuals plotted against theoretical quantiles (Q–Q plot), (top-right) residuals plotted against linear predictors, (bottom-left) histogram with frequency of residuals) and (bottom right) response plotted against fitted values. Table S2: Coefficients of parametric and smooth terms, for global model and *I. ricinus* nymph model. Significance codes: 0 ‘ ^*∗∗∗*^', 0.001 ‘ ^*∗∗*^', 0.01 ‘ ^*∗*^' and 0.05.
