# Supplementary material for: An Integrated Approach to an Emerging Problem: Implementing a Whole Year of Camera Trap Survey in Evaluating the Impact of Wildlife on Tick Abundance
Source: Transbound Emerg Dis. 2024 Sep 26;2024:4064855. doi: 10.1155/2024/4064855 (PMC12016756; doi:10.1155/2024/4064855)
Supplement: Supporting Information — Figure S1: Temporal trend of ticks collected in La Mandria. The graph shows the trends per developmental stage and species, plotting the logarithmic mean number over the month of collection. The red line defines all species together, while single species are plotted with thinner lines. Table S1: AIC model selection. Significance codes: p=0 ‘ ∗∗∗', p=0.001 ‘ ∗∗',p=0.01 ‘ ∗' and p=0.05‘.'. The k value is the smoothing parameters and determines how many basis functions are used to approximate the smooth function for each predictor. The cubic regression splines (‘cc') were used as basis function (bs). Figure S2: Residual analysis of the final GAM global model (A) and I. ricinus nymph model (B). We plot the following: (top-left) deviance residuals plotted against theoretical quantiles (Q–Q plot), (top-right) residuals plotted against linear predictors, (bottom-left) histogram with frequency of residuals) and (bottom right) response plotted against fitted values. Table S2: Coefficients of parametric and smooth terms, for global model and I. ricinus nymph model. Significance codes: 0 ‘ ∗∗∗', 0.001 ‘ ∗∗', 0.01 ‘ ∗' and 0.05. [file 4064855.f1.docx]

Supplementary Figure 1: Temporal trend of ticks collected in La Mandria. The graph shows the trends per developmental stage and species, plotting the logarithmic mean number over the month of collection. The red line defines all species together, while single species are plotted with thinner lines.


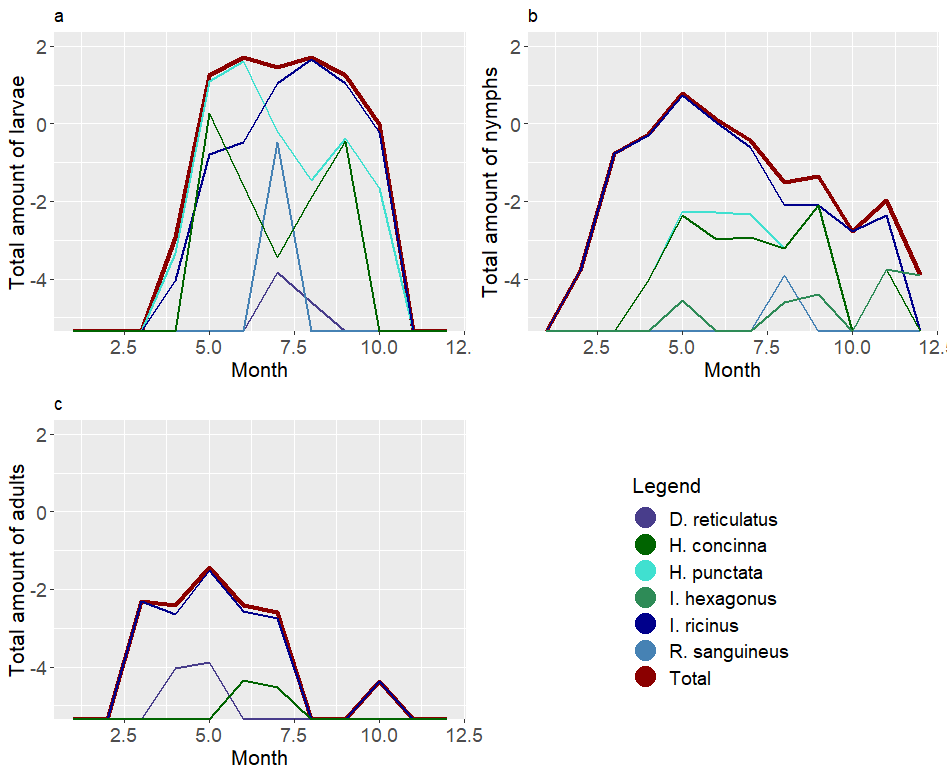


Supplementary Table 1: AIC model selection. Significancy codes codes: *p* = 0 ‘***’, *p* = 0.001 ‘**’, *p* = 0.01 ‘*’, *p* = 0.05 ‘.’ . k value is the smoothing parameters and determines how many basis functions are used to approximate the smooth function for each predictor. The cubic regression splines (“cc”) was used as basis function (bs).

| Model | AIC |
| --- | --- |
| Mesocarnivores_TO+ WildBoar_TO + WildRuminants_TO | 30830.71 |
| SmaxSD1+NDVI + Month + Mesocarnivores_TO + WildBoar_TO + WildRuminants_TO | 30487.80 |
| SmaxSD1*NDVI + s(Month, bs="cc", k=6) + Mesocarnivores_TO + WildBoar_TO + WildRuminants_TO | 25653.16 |
| SmaxSD1 + NDVI + s(Month, bs="cc", k=6) + s(Mesocarnivores_TO, k=3) + s(WildBoar_TO, k=3) + s(WildRuminants_TO, k=3) | 25335.71 |
| SmaxSD1 + NDVI + s(Month, bs="cc", k=6) + s(Mesocarnivores_TO, Season, k=3) + s(WildBoar_TO, Season, k=3) + s(WildRuminants_TO, Season, k=3) | 25322.41 |

Supplementary Figure 2: residual analysis of the final GAM global model (A) and *I. ricinus* nymphs model (B). We plot: (top-left) deviance residuals plotted against theoretical quantiles (Q-Q plot), (top-right) residuals plotted against linear predictors, (bottom-left) histogram with frequency of residuals), (bottom right) response plotted against fitted values.


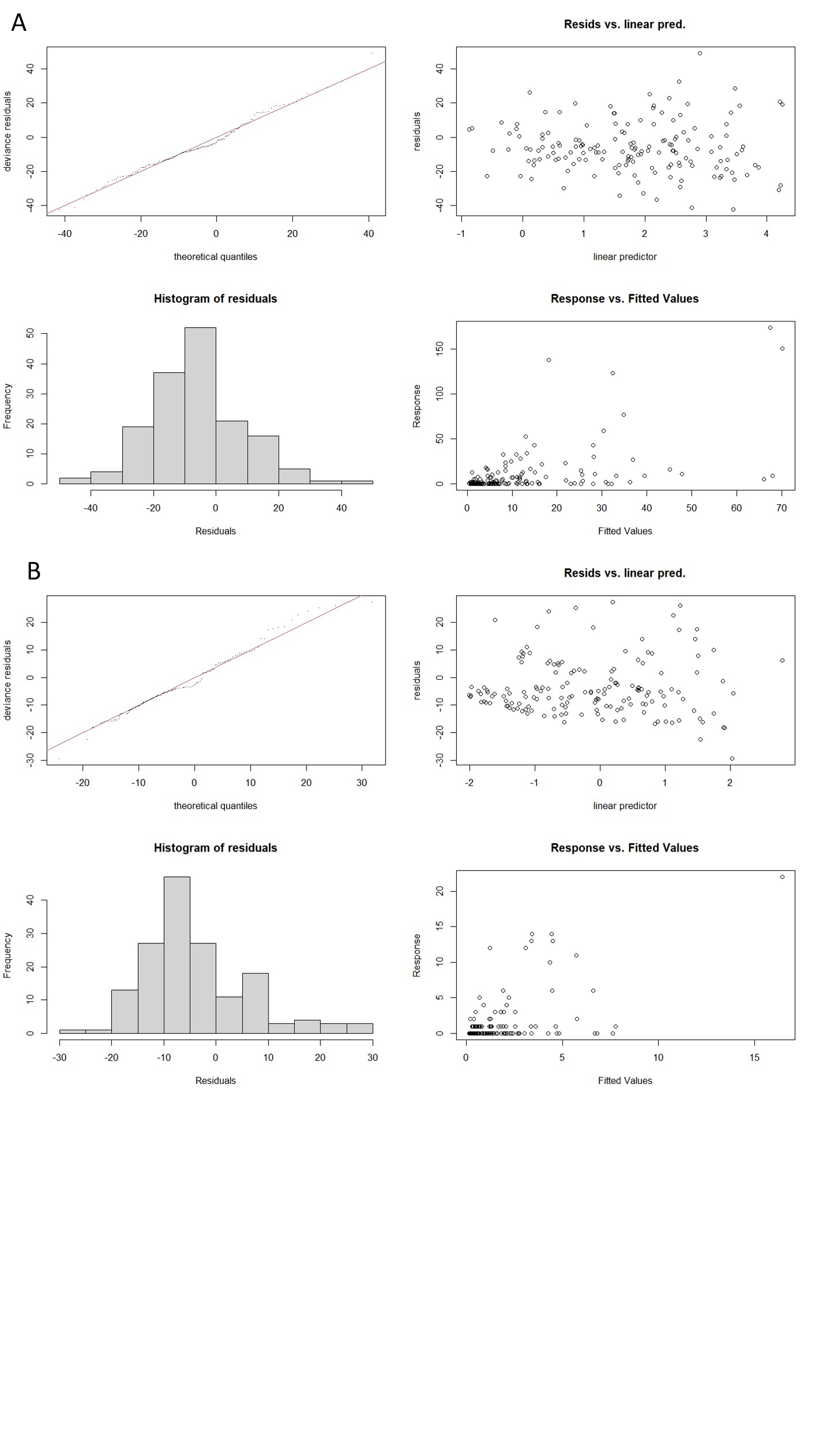


Supplementary table 2: Coefficients of parametric and smooth terms, for global model and *I. ricinus* nymphs model. Signif. codes: 0 ‘***’ 0.001 ‘**’ 0.01 ‘*’ 0.05

| **Global model** |  | | | |  | |  | |  |  |
| --- | --- | --- | --- | --- | --- | --- | --- | --- | --- | --- |
| Parametric coefficients: | | | | | | | | | |  |
|  | Estimate | Std. Error | t value | Pr(>\|t\|) | |  | | | | |
| (Intercept) | 1.80786 | 0.01918 | 94.252 | <2e-16 | | *** | |  |  |  |
| maxSD | -0.04212 | 0.01909 | -2.207 | 0.0289 | | * | |  |  |  |
| NDVI_scaled | -0.62611 | 0.02208 | -28.353 | <2e-16 | | *** | |  |  |  |
| Approximate significance of smooth terms: | | | | | | | |  |  |  |
|  | edf | Ref.df | F | p-value | |  | |  |  |  |
| s(Month) | 3.991 | 4 | 1088.5 | <2e-16 | | *** | |  |  |  |
| s(Mesocarnivores_TO,Season) | 2.972 | 2.999 | 103.2 | <2e-16 | | *** | |  |  |  |
| s(Wildboar_TO,Season) | 1.994 | 2 | 330 | <2e-16 | | *** | |  |  |  |
| s(WildRuminants_TO,Season) | 1.959 | 1.998 | 236.8 | <2e-16 | | *** | |  |  |  |
| ***I. ricinus* nymphs model** |  |  |  |  | |  | |  |  |  |
| Parametric coefficients: | | | | | | | |  |  |  |
|  | Estimate | Std. Error | t value | Pr(>\|t\|) | |  | |  |  |  |
| (Intercept) | -0.13786 | 0.02084 | -6.615 | 7E-10 | | *** | |  |  |  |
| maxSD | 0.13478 | 0.0229 | 5.885 | 3E-08 | | *** | |  |  |  |
| NDVI_scaled | -0.45545 | 0.02376 | -19.169 | <0 | | *** | |  |  |  |
| Approximate significance of smooth terms: | | | |  | |  | |  |  |  |
|  | edf | Ref.df | F | p-value | |  | |  |  |  |
| s(Month) | 3.969 | 4 | 645.52 | <2e-16 | | *** | |  |  |  |
| s(Mesocarnivores_TO,Season) | 2.002 | 2.004 | 75.34 | <2e-16 | | *** | |  |  |  |
| s(Wildboar_TO,Season) | 1.994 | 2 | 214.35 | <2e-16 | | *** | |  |  |  |
| s(WildRuminants_TO,Season) | 1.005 | 1.009 | 71.57 | <2e-16 | | *** | |  |  |  |
